# Beyond the chaos: How architecture structures tumour biology

**DOI:** 10.1111/febs.70470

**Published:** 2026-02-22

**Authors:** Lea Dörner, Catrin Lutz, Stefan Prekovic, Hendrik A. Messal

**Affiliations:** ^1^ Division of Tumor Biology and Immunology The Netherlands Cancer Institute Amsterdam The Netherlands; ^2^ Center for Molecular Medicine UMC Utrecht The Netherlands

**Keywords:** biochemistry, biophysics, cellular neighbourhoods, microenvironment, Tissue architecture, tumour biology

## Abstract

Cancer is increasingly recognised as a complex and heterogeneous disease, shaped not only by genetic mutations but also by the physical and biochemical context in which tumours develop. The spatial position of a cell, including its physical, cellular and molecular surroundings, shapes its fate, phenotypic plasticity and potential to transform and drive tumour progression and evolution. Tissue architecture provides a powerful framework for understanding the complex dynamics of cancer. It integrates the structural organisation of the tumour and its surrounding tissue, the distribution of physical forces, biochemical niches, cellular neighbourhoods, and the broader tissue and organ context in which the tumour develops. Together, these elements form a dynamic and evolving landscape that is continuously remodelled through the multiscale communication of cellular, biochemical and mechanical components. Understanding the principles that govern these interactions reveals that cancer is not merely a chaotic aggregation of cells, but a patterned system shaped by coordinated spatial relationships. Here, we discuss the recent literature to examine how physical, biochemical and cellular relationships orchestrate tumour initiation, progression and treatment resistance, and how their collaboration acts not as a passive scaffold, but as the architect of tumour behaviour.

## Graphical abstract

Tissue architecture shapes tumour initiation and progression through multiple interconnected layers continuously remodelled over time. This review outlines how physical forces, biochemical cues, cellular niches and systemic influences contribute to tumour evolution. We describe how these layers promote or restrain transformation during initiation within physiological architecture, how they change as tumours progress and how dynamic, multi‐directional crosstalk between these layers guides cancer behaviour, offering a structured perspective on tumour complexity.

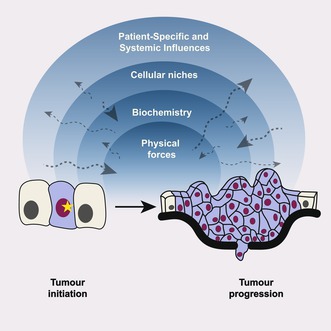

**Abbreviations**
BCC: basal cell carcinoma
BM: basement membrane
CAF: cancer‐associated fibroblast
CN: cellular neighbourhoods
CRC: colorectal cancer
ECM: extracellular matrix
EMT: epithelial‐to‐mesenchymal transition
IFN: interferon
IFP: interstitial fluid pressure
IL: interleukin
NOTCH: notch homologue protein
PIEZO: piezo type mechanosensitive ion channel component
SCC: squamous cell carcinoma
TCR: T‐cell receptor
TGF: transforming growth factor
TLS: tertiary lymphoid structures
TME: tumour microenvironment
TNF: tumour necrosis factor
VEGF: vascular endothelial growth factor

## Introduction

Recent advances in oncology have revealed cancer as a complex, heterogeneous disease shaped by both cell‐intrinsic factors, such as genetic mutations, and extrinsic influences from the microenvironment, including physical, biochemical and cellular interactions [1, 2, 3]. While oncogenic mutations are essential for cancer initiation, they are common in normal human tissues. DNA is continually exposed to damage throughout life, and despite effective repair mechanisms, this leads to the accumulation of driver mutations in bona fide cancer genes [4, 5, 6, 7]. Sequencing of sun‐exposed eyelid skin revealed that 18–32% of cells carried mutations in cancer‐related genes, such as neurogenic locus notch homologue protein 1 (NOTCH1), translating to roughly 140 oncogenic mutations per cm^2^ of normal skin [7]. Likewise, a 2021 study estimated that cancer‐free elderly individuals harbour over 100 billion cells containing one or more oncogenic mutations [6]. While cancer is widely recognised as a multiple‐hit disease [8, 9], requiring the accumulation of several cooperating genetic alterations, these findings highlight a key paradox: even when such mutations are widespread in normal tissues, cancer remains relatively rare, indicating that additional factors may be required for tumour development.

In the 1940s, Isaac Berenblum proposed that genetic mutations alone are insufficient for tumour formation, introducing the concept of a necessary ‘promoting process’ [10, 11]. Subsequent studies have confirmed that tumour development is not only influenced by genetic alterations but also by the biochemical and structural milieu in which the mutations occur, including hormones delivered through the vasculature, growth factors from the mesenchyme, signals from neighbouring wild‐type cells and environmental components such as the extracellular matrix (ECM), cancer‐associated fibroblasts (CAFs) and immune cells [1, 12, 13, 14, 15, 16, 17, 18]. Together, the cells and structural components surrounding transformed cells constitute the tumour microenvironment (TME) [1], which in this review, rather than being treated as a uniform entity, will be dissected into its constituent parts in the broader concept of tissue architecture.

Tissue architecture refers to the structural and functional organisation of tissues, encompassing physical forces, biochemical gradients, cellular heterogeneity and spatial configuration, all contextualised within the native tissue where a tumour arises [3]. This architecture is dynamic in both health and disease, involving continuous interactions across multiple scales [19]. Physical forces emerge from whole‐organ mechanics, tumour growth, ECM remodelling and intrinsic cellular properties, such as stiffness, contractility and cytoskeletal organisation [3, 20]. Biochemical gradients also span several orders of magnitude, ranging from hormones and growth factors delivered via vasculature to local diffusion of cytokines, nutrients and metabolic by‐products [21, 22, 23]. Crucially, tissue architecture is not a passive result of cellular activity, but an active framework that shapes behaviour: mechanical cues trigger mechanotransduction and biochemical gradients establish gene modulatory niches, governing phenotypic plasticity and multi cell type crosstalk in cellular neighbourhoods. These interactions create feedback loops that operate from subcellular to tissue‐wide levels, ensuring no component functions in isolation [18, 23, 24], a multiscale dynamism particularly relevant in the context of antitumour immunity [25, 26, 27, 28].

In this review, we explore the diverse components of tissue architecture, moving from physical forces to biochemical interactions and cellular crosstalk, and discuss their interwoven roles in tumour initiation, progression and immune modulation. By viewing cancer as a patterned and coordinated system, rather than a disordered cell mass, we aim to refine our understanding of its structured dynamics and their relevance for therapy development, a growing notion increasingly recognised in recent research [29].

## Physical forces and mechanobiology

### Physical forces in a biological context

**Fig. 1:**
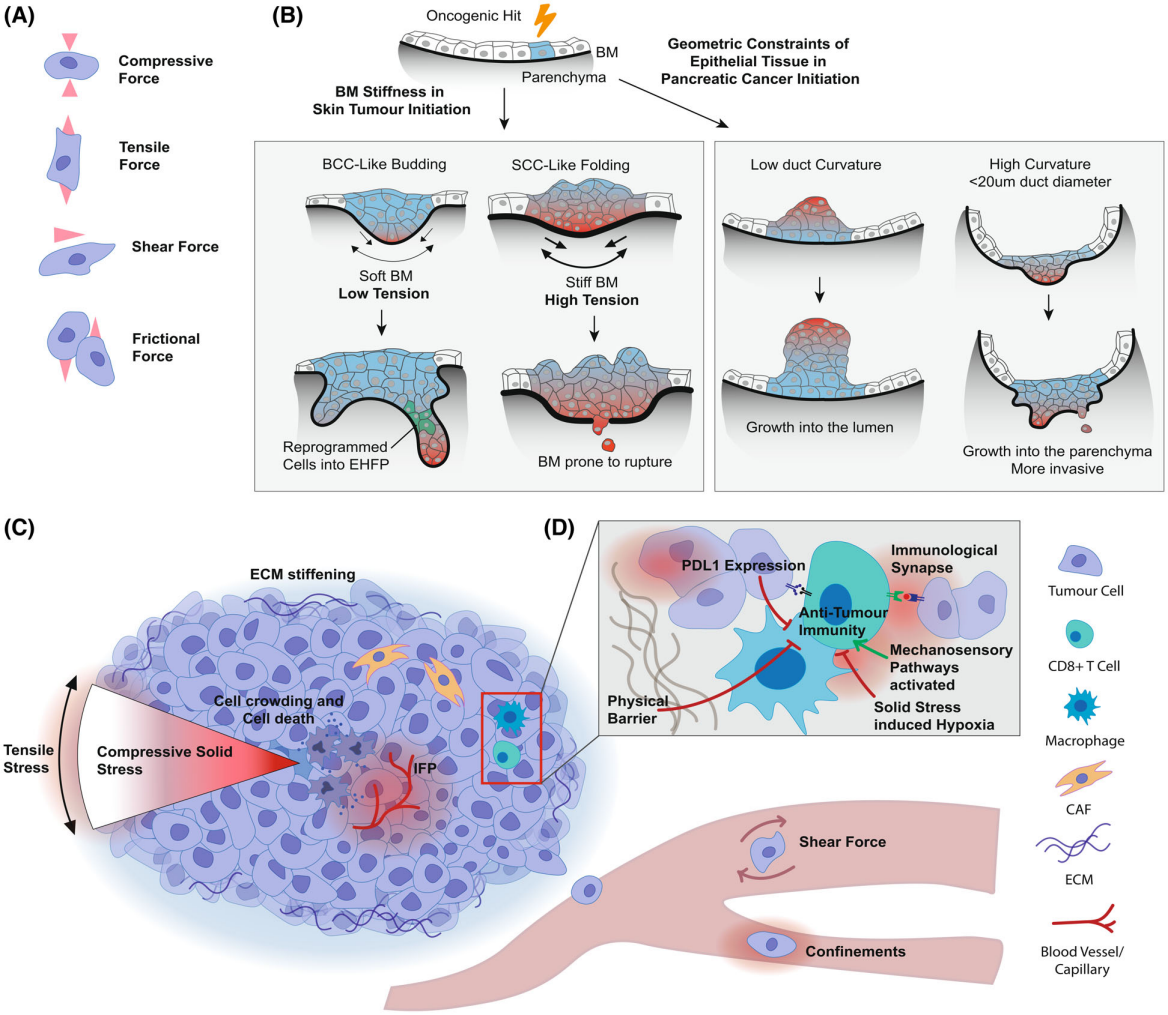
Physical forces during tumour initiation and progression. (A) Physical forces acting on cells within tissues, with red arrows indicating the direction and axis of force application. (B) BM stiffness and geometry of the tissue of origin influence tumour initiation and the morphology of lesions. (C) Illustrative overview of physical forces acting on cells in a progressed tumour. (D) Zoom in on the effects of physical forces in a progressed tumour on antitumour immunity, where red arrows indicate negative effects and green arrows indicate positive effects on immune cell infiltration, activation and function. BCC, Basal cell carcinoma; BM, basement membrane; CAF, cancer‐associated fibroblast; ECM, extracellular matrix; EHFP, embryonic hair follicle progenitor; IFP, interstitial fluid pressure; SCC, squamous cell carcinoma.

The mechanical properties of individual cells and their surrounding environment collectively shape the physical forces acting within tissues [20]. In cancer, these mechanical properties and forces influence tumour initiation and evolve during progression [2, 3, 30, 31, 32]. These forces act and interact across spatial scales, from the subcellular and cellular level to the tissue and organ level, influencing both individual cell behaviour and collective tissue dynamics. The primary forces acting on cells within biological tissues include tensile, compressive, shear and frictional forces (Fig. 1A). Tensile forces stretch and elongate cells along their axes, while compressive forces push or compress cells. Shear forces arise from fluid movement, such as blood flow or interstitial fluid dynamics, acting parallel to the cell surface. Frictional forces occur when two surfaces interact, resisting relative motion and acting parallel to the contact surfaces [20, 33].

As cells respond to forces, they engage mechanotransduction, which converts mechanical signals into biochemical responses and enables environmental adaptation [34, 35]. The cytoskeleton, composed of actin filaments, microtubules and intermediate filaments, governs key mechanical properties, such as elasticity, contractility and membrane tension [36]. It not only provides structural support but also plays a central role in mechanotransduction, enabling cells to convert physical stimuli into biochemical signals [20, 37]. Mechanosensitive ion channels, such as transient receptor potential cation channel subfamily V member 4 (TRPV4) and piezo type mechanosensitive ion channel component (PIEZO), detect membrane deformation, triggering calcium influx and activating downstream signalling pathways [24]. In parallel, mechanotransduction pathways, such as Hippo respond to changes in cell shape and adhesion, and link mechanical signals with cell behavioural cues through regulating proliferation and apoptosis [38].

Cell perception and response to mechanical cues are influenced by both the cell‐intrinsic mechanical characteristics, primarily dictated by the cytoskeleton, and the properties of the surrounding environment, including geometry, fluid dynamics and stiffness. For example, the actin cortex regulates surface and line tensions along the plasma membrane, directly influencing cell shape and internal tension. Microtubules resist compression, while intermediate filaments contribute to overall elasticity [35, 39]. Additionally, cell–cell adhesion mediated by cadherins and selectins, and cell–ECM adhesion via integrins balance internal forces and facilitate force transmission between cells [30]. Furthermore, cells can actively generate traction forces by contracting their actomyosin machinery, pulling against the ECM through focal adhesions where integrins anchor the cytoskeleton to the matrix [34, 39].

### Mechanobiology of tumour initiation

The interplay of physical forces resulting from the mechanical properties of individual cells and their surrounding environment significantly influences tumour initiation. Stiffness of the environment, quantitatively described by the elastic modulus, which measures a material's resistance to deformation, is determined primarily by ECM composition, organisation and density [34, 40], which vary intrinsically between tissues, shaping their mechanical and functional properties [41]. At the molecular level, matrix stiffness depends on the balance between the deposition and degradation of ECM components, such as fibrillar collagens, fibronectin isoforms, tenascin‐C, osteopontin, osteonectin and periostin [2, 42, 43]. In addition, enzymatic crosslinking of ECM proteins, such as collagen and elastin by lysyl oxidases (LOX) and transglutaminases strengthens the matrix and elevates tissue rigidity [2].

A stiff ECM impairs epithelial defence against cancer by disrupting the cytoskeletal dynamics required to extrude transformed cells. Under normal, soft ECM conditions, the cytoskeletal protein filamin localises to the interface between normal and transformed cells, where it contributes to cell extrusion. However, increased ECM stiffness causes filamin to accumulate perinuclearly, preventing its redistribution to the cell–cell interface and thereby weakening the mechanical forces required for extrusion, ultimately compromising epithelial defence against cancer efficacy [44]. The stiffness of the basement membrane (BM) also influences tumour morphology during initiation. Noninvasive basal cell carcinoma (BCC), for example, typically forms bud‐like structures within softer BMs, which can accommodate tensile forces. In contrast, squamous cell carcinoma (SCC) forms fold‐like structures associated with stiffer BMs that resist deformation, facilitating rupture and cancer cell invasion [45, 46]. In this context, a recent study showed that oncogenic SmoM2‐mutant cells could expand into bud‐like structures within a softer ECM, generating mechanical tension in the surrounding epithelium and reprogramming interfollicular cells into embryonic hair follicle progenitor states necessary for BCC initiation. In stiffer ECMs, such as the skin of the back, these processes were inhibited, preventing vertical growth transitions and tumour initiation (Fig. 1B) [46].

Tissue geometry, the three‐dimensional shape, size and configuration of cells and tissues, emerges from the interplay and balance of physical forces [47]. Geometry shapes the distribution of tension and compression and can directly influence tumour initiation [48]. In pancreatic ducts, transformed cells lose the normal apical‐to‐basal gradient of myosin activity, altering their surface tension and favouring inward growth into the ductal lumen. However, in small‐diameter ducts, the cells' basal surface area is larger due to the higher curvature needed to form a narrow cylindrical epithelium. This geometry requires relatively greater basal myosin contractility to constrict the basal side enough for inward growth. If this contractile force is insufficient, the cells instead grow outward into the surrounding parenchyma. Such early differences in tumour growth patterns can have lasting effects on tumour biology; for instance, in small pancreatic ducts, outward expansion into the surrounding parenchyma increased the tumour–stroma interface, which was associated with enhanced recruitment of CAFs [48]. Thus, tissue‐level mechanical properties and geometry can shape cellular decisions, influencing tumour initiation, morphology and even predisposition to invasiveness (Fig. 1B) [45, 48].

### Physical forces evolve during tumour progression

As solid tumours progress, evolving cellular and extracellular structures reconfigure existing physical forces, altering the mechanical landscape of the TME [32, 49]. Tumour cells, CAFs and other TME components remodel the ECM, increasing its stiffness and promoting tumour expansion by exerting pressure on adjacent tissues (Fig. 1C) [2, 20, 50].

Solid stress emerges from the accumulation of nonfluid tumour components and the mechanical constraints of surrounding tissue. It manifests as outward tensile forces at the tumour margins and compressive stress within the core. This stress varies by tumour type, from 0.21 kPa in brain tumours to over 7 kPa in highly desmoplastic pancreatic tumours [51]. Compressive forces impair vascular and lymphatic function, leading to interstitial fluid pressure (IFP), a hydrostatic force acting isotropically, further exacerbating physical confinement (Fig. 1C) [52, 53].

These forces influence tumour cells differently depending on magnitude and tumour stage. In the intestine, tumour pressure promotes β‐catenin phosphorylation, disrupting E‐cadherin binding and enhancing apical localisation, which drives proliferation and activates oncogenic programmes like myelocytomatosis (MYC) [54]. ECM stiffness, solid stress and elevated IFP have all been shown to enhance cell migration, epithelial‐to‐mesenchymal transition (EMT) and invasiveness [55, 56, 57]. Physical confinement also deforms nuclei, leading to epigenetic alterations and DNA damage that can promote invasive behaviour [58, 59, 60]. For instance, recent work revealed that mechanical forces can directly remodel tumour‐cell chromatin to sustain invasive phenotypes. In melanoma, confinement‐induced mechanical force exerted on the cell upregulates the chromatin‐associated protein High Mobility Group Box 2 (HMGB2), which increases chromatin accessibility at invasion‐related loci, thereby epigenetically stabilising the invasive state [61]. However, when solid stress exceeds critical thresholds, it can induce apoptosis or necrosis, suppressing tumour growth [62].

During tumour progression, tumour cells face ongoing physical challenges, including mechanical constriction during intra‐ and extravasation and fluid shear stress in circulation. These different biophysical environments may contribute to tumour‐cell clearance, leaving only a subset capable of seeding metastases. Notably, the different organ contexts of the metastatic sites again represent environments that differ biomechanically from the primary tumour site [63, 64, 65].

### Mechanobiology modulates tumour immunology

Physical forces in tumours critically influence antitumour immunity [66]. Tumours with increased ECM stiffness and solid stress were found to frequently exhibit a reduced immune cell density within their core and an overall immunosuppressive environment, in contrast to softer tumours that are more permissive to immune cell infiltration [67]. The complex relationship between mechanical forces and immune function is exemplified by physical cues that modulate immune cell activation, often lowering activation thresholds via mechanosensory pathways [68]. For example, the mechanosensory Hippo pathway, in crosstalk with rat sarcoma viral oncogene homologue (Ras) signalling, activates genes promoting T‐cell immunity [69]. Mice with T cells lacking the mechanosensitive ion channel PIEZO1 show reduced antitumour responses [70]. In dendritic cells, PIEZO1 supports antitumour immunity by promoting T helper 1 (TH1) differentiation while suppressing regulatory T‐cell (T_reg_) development [71]. Likewise, activation of mechanosensory pathways in macrophages has been shown to enhance polarisation towards a pro‐inflammatory phenotype [72, 73].

Nevertheless, the mechanical properties of the TME can also hinder immune function (Fig. 1D). Dense ECM and solid stress present physical barriers to immune cell infiltration and restrict motility. Leukocytes, especially T cells, rely on amoeboid migration, which involves actomyosin‐driven shape changes and pseudopodia formation [74]. In denser ECMs, their migration slows due to smaller pore sizes and increased resistance, requiring immune cells to exert greater force to deform or navigate through the matrix. Nuclear deformability often becomes a limiting factor in such confined spaces [27, 28].

Importantly, mechanical forces affect the formation and stability of the immunological synapse between cytotoxic lymphocytes and tumour cells. T cells exert forces, such as actin retrograde flow, to stabilise interactions with antigen‐presenting cells. When mechanical load remains below a critical threshold, T‐cell receptor–peptide–major histocompatibility complex (TCR–pMHC) bond lifetimes are extended, enhancing signalling. Once this threshold is exceeded, these bonds convert into slip bonds, accelerating dissociation and weakening TCR signalling [75]. This suggests that while the activation of mechanosensory pathways in immune cells generally lowers their threshold for activation and while force can even enhance immunological synapse formation, the solid stress present in many solid tumours often exceeds this threshold, hindering immune cell trafficking and impairing immunological synapse formation (Fig. 1D).

In addition to these direct effects, mechanical stress and ECM stiffness have been shown to increase programmed death‐ligand 1 (PD‐L1) expression on tumour cells, further promoting immune evasion by dampening T‐cell responses through checkpoint engagement (Fig. 1D) [76, 77, 78, 79]. Moreover, mechanical compression of blood and lymphatic vessels may create an unfavourable biochemical landscape by limiting perfusion, impairing nutrient and oxygen delivery, and exacerbating hypoxia, all of which contribute to reduced immune surveillance [20, 52].

Together, physical forces represent a critical dimension of tissue architecture, shaping tumour dynamics from initiation to metastasis. The evolving mechanical landscape of tumours influences cell behaviour, immune responses and metastatic efficiency. Understanding how these forces emerge and act across spatial scales, from the subcellular to the tissue level, is essential for decoding tumour biology. More work needs to be carried out to explore how mechanical forces structure the biochemical landscape of tumours, through modulating soluble cues, molecular gradients and tissue‐specific metabolic environments, and how this influences tumour behaviour and immune responses.

## Biochemistry

### Biochemistry in the context of tissue architecture

The interplay between physical forces and biochemical signals is fundamental to tissue architecture, with mechanical cues shaping intracellular molecular programmes, tissue molecular gradients, and vice versa, jointly orchestrating cellular behaviour in both healthy and tumour contexts [18, 80, 81, 82, 83, 84]. Biochemical signals are spatially and contextually organised within tissue‐specific molecular environments, shaped by oxygen and metabolite availability, cytokine gradients and the composition and structure of the ECM. This biochemical landscape profoundly influences cell behaviour and fate, enabling context‐specific responses to the unique combinations of stimuli that characterise each microenvironment [18, 80, 81]. Alterations to this environment, whether due to physiological states, such as hormonal cycles or pathological conditions like chronic inflammation or obesity, can affect mutated cells and increase their likelihood of progressing towards a tumorigenic state [14, 15, 85, 86, 87].

As tumours progress, regional biochemical heterogeneity increases, driven by cellular diversity and spatial variation in metabolism, secretion and consumption of soluble factors, and ECM remodelling [21, 22, 23]. This diversity is compounded by the varied signalling ranges of endocrine, paracrine and autocrine factors [88]. For instance, hormones circulate systemically, while molecules, such as vascular endothelial growth factor (VEGF) act locally, and autocrine factors remain confined to the producing cell. Signal distribution is further shaped by diffusion properties, molecular stability and the nature of the surrounding environment, including ECM density and porosity [80, 81, 89, 90, 91].

A growing tumour spans multi‐scale signalling ranges, defined by gradients that may cover the entire tumour mass or remain highly localised and create biochemical niches. These spatially organised signals are not isolated but integrate with physical cues and cellular neighbourhoods to influence cell fate and immune responses. This section explores how biochemical features shape and are shaped by tissue architecture, with implications across tumour initiation, progression and immunity.

### Biochemical landscape in tumour initiation

Cellular tumour initiating potential is engrained in cell‐intrinsic biochemistry through subcellular organisation and metabolic pathways that feed into the mechanisms governing cell‐fate integrity and transformation [92, 93]. In addition to the cell‐intrinsic molecular make‐up, the physiological biochemical landscape surrounding cells, instructive for maintaining epithelial integrity and function in tissue homeostasis, influences the behaviour of mutated cells and their progression towards a tumorigenic state. In intestinal crypts, gradients of WNT, NOTCH and EPH/Ephrin maintain the stem cell niche vital for the epithelial turnover in the harsh environment of the gut [94]. WNT ligands secreted by mesenchymal and epithelial cells create a gradient that peaks at the crypt base, stimulating intestinal stem cell proliferation, while declining WNT levels towards the villus promote differentiation. NOTCH signalling maintains intestinal stem cell multipotency and guides cell‐fate decisions. Within this setting, intestinal stem cells compete through neutral drift [95]. Mutations that confer autonomy from the regulatory biochemical milieu provide a fitness advantage, allowing a clone to dominate the niche and predisposing the crypt to oncogenic transformation [94, 96].

Hormones play a prominent role among the biochemical factors influencing tumorigenesis. Tissues with intrinsic hormonal activity are shaped by their biochemical milieu in ways that affect their susceptibility to malignant transformation. In the prostate, androgens activate the androgen receptor, while in breast, ovarian and endometrial tissues, oestrogens act via oestrogen receptors, such as ERα [14, 15]. While these hormones support differentiation under physiological conditions, their signalling pathways may be hijacked by oncogenic alterations or upstream regulators, decoupling them from their normal differentiation roles and reprogramming them to support proliferation and survival, thereby accelerating tumour initiation [14, 15]. In the mammary epithelium, oestrogen fluctuations during the oestrous cycle drive oscillating waves of proliferation and apoptosis. This process acts both as a protective and accelerating factor in mammary tumorigenesis. While somatic mutations have been shown to be frequently eliminated during oestrous‐driven mammary remodelling, surviving clones may expand drastically, propagating mutations over large distances in the mammary ductal trees [87]. This can predispose large fields to transformation and offers an explanation for the phenomenon of the ‘sick lobe’ which proposes that certain breast lobes are inherently more prone to transformation [87, 97, 98]. In support of this theory, precancerous alterations have been found in morphologically normal ducts adjacent to ductal carcinoma *in situ* indicating the presence of predisposed fields in the human breast [99].

The role of oestrogen in oncogenesis extends beyond promoting proliferation to directly affecting genomic stability via the mutational process of translocation‐bridge amplification [86]. This mechanism induces early focal oncogene amplifications, including human epidermal growth factor receptor 2 (*EGFR2*) and Cyclin D1 (*CCND1*), which define clinically relevant breast cancer subgroups [14, 100]. While the broader impact of oestrogen on cancer genomes remains to be fully characterised, pan‐cancer analyses suggest that translocation‐bridge amplifications are common across cancers, with a modestly higher prevalence in women [86].

Hence, the biochemical landscape of healthy tissues influences tumour initiation by modulating the behaviour and fate of mutated cells. Tissue‐specific biochemical signals can amplify or suppress the oncogenic potential of mutations, ultimately shaping whether such mutations remain silent or lead to malignant transformation.

### Biochemical heterogeneity modulates tumour progression

Tissue‐specific biochemical cues remain instructive during tumour progression. Alterations in upstream regulators of hormone receptor signalling in breast and prostate cancers reflect a lasting dependency on oestrogen and androgen signalling, respectively. This biochemical ‘addiction’ to specific oncogenic pathways persists throughout tumour evolution, a vulnerability long exploited through targeted treatments [14, 15].

Beyond tumour‐cell‐derived factors, the biochemical environment is further shaped by signals from stromal components, including fibroblasts and macrophages. Their secretion of factors like VEGF and basic fibroblast growth factor (bFGF), common across many tissues, interacts with local differences in cellular composition and ECM context, which together shape regional variations in angiogenesis and vascular architecture, including vessel cohesiveness, pericyte coverage and adhesion molecule expression [101, 102, 103, 104].

As tumours grow, their internal biochemical heterogeneity intensifies. This is reflected in uneven distributions of signalling molecules, nutrients, oxygen and ECM components. The ECM, composed of proteins, glycoproteins and polysaccharides, is continuously remodelled by tumour and stromal cells [2, 34]. Besides determining mechanical properties like stiffness [27, 28, 34, 40, 74], the ECM also directly modulates immune responses. For example, collagens, including both transmembrane and secreted forms like collagen type I, serve as high‐affinity ligands for leukocyte‐associated immunoglobulin‐like receptor 1 (LAIR‐1), an inhibitory receptor on CD4+ and CD8+ T cells, attenuating the cytotoxic activity of natural killer cells and contributing to immune evasion in stiff tumours [105, 106].

**Fig. 2:**
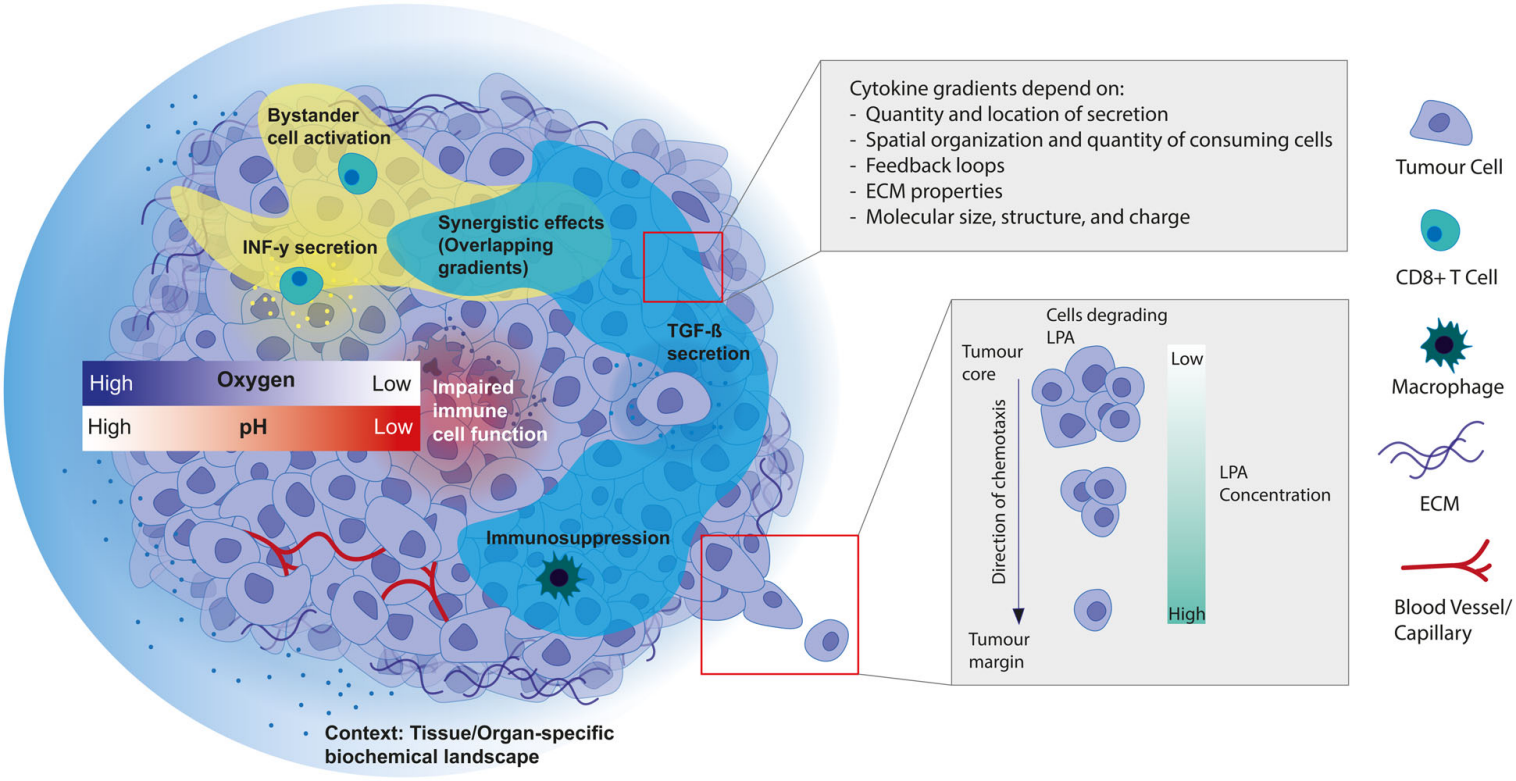
Biochemical landscape of a solid tumour. Simplified overview of the biochemical landscape in progressing tumours. The figure illustrates how tissue of origin, ECM composition and organisation, oxygen, pH and soluble molecule gradients collectively shape the tumour biochemical landscape and influence cellular behaviour within it. ECM, extracellular matrix; IFN‐γ, Interferon‐gamma; LPA, lysophosphatidic acid; pH, potential of hydrogen; TGF‐ß, Transforming growth factor‐β.

Another aspect of biochemical variability in tumours is the supply of nutrients and oxygen, constrained by diffusion processes, limiting the effective support of cells to within 100–200 μm of the nearest capillary due to the high cellular demand and rapid uptake of oxygen for respiration [107]. Overcoming these limits, tumours induce angiogenesis, yet solid stress and imbalances in angiogenic regulators lead to abnormal blood vessels and chaotic blood flow, creating regions with varying oxygen levels and hypoxia (Fig. 2) [22]. Hypoxia modulates tumour and TME cell behaviour and impairs immune function by altering cell signalling and limiting effector activity. It drives metabolic reprogramming towards aerobic glycolysis (the Warburg effect), resulting in increased lactic acid production and extracellular acidification [20, 22, 108, 109]. This creates pH variability across the tumour and can result in a lower pH at the tumour core (Fig. 2) [20, 110, 111]. Extracellular acidification weakens cell–cell adhesion and promotes matrix adhesion, which is suspected to synergistically promote metastasis by facilitating the detachment of single cells from the tumour epithelium and enabling their invasion into the surrounding tissue [112].

### Biochemical gradients shape regional tumour diversification

In addition to local heterogeneities in metabolites and catabolites, molecular diffusion shapes the complex biochemical landscape by creating gradients of signalling molecules modulating cellular behaviour [88]. Tumour cells utilise these gradients as steering cues for invasion into surrounding tissues [23]. Their directional movement, or chemotaxis, relies on differences in attractant concentration detected by surface receptors, with steeper gradients improving efficiency [113]. Cells respond to attractant concentrations within a specific range: low levels produce gradients that are too shallow to detect, while high concentrations saturate receptors, impairing spatial perception. Tumour cells can generate self‐imposed gradients by locally degrading attractants, thereby producing steep concentration differences that guide long‐range migration [113, 114, 115, 116]. For instance, melanoma cells establish chemotactic gradients from a uniformly distributed exogenous supply of lysophosphatidic acid (LPA) [117]. This process relies on the lipid phosphatase LPP3, which degrades extracellular LPA [118], leading to areas of higher cell density with reduced local attractant concentrations. Such local depletion creates outward‐facing gradients, encouraging cell dispersal and directly promoting dissemination (Fig. 2). Notably, while not contributing to directional specificity, gradients of epidermal growth factor and platelet‐derived growth factor, as well as their combination, accelerated the speed of chemotaxis towards LPA [117]. Such dynamic gradient shaping allows cells to avoid dead ends and navigate complex environments [115, 116].

### Cytokine gradients modulate tumour‐immune cell interactions

Cytokine gradient formation is modulated by tumour‐specific factors, such as ECM density, acidosis and fibre alignment [91, 110, 119]. The charge of ECM proteins, modulated by posttranslational modifications, such as their decoration with glycosaminoglycans, determines their freedom to interact with and sequester cytokines whose net charge is context‐dependent, varying with pH and structural conformation [119, 120, 121].

Cytokines form diffusion gradients that define immunological niches, varying in range and concentration (Fig. 2). Interferon‐gamma (IFN‐γ) can signal across hundreds of micrometres in tumours [122], although the effective diffusion range is likely to vary significantly with tissue context, cellular density and ECM composition, whereas others like Interleukin 4 (IL‐4) generate steep, short‐range gradients shaped by ECM architecture and local consumption [80, 91, 119]. Such cytokine fields are not static but dynamic, responding to fluctuations in cytokine production and consumption. For example, feedback loops involving IL‐4 and IFN‐γ can amplify cytokine production and promote the differentiation of bystander T cells into cytokine‐producing subpopulations [123, 124]. In reactive lymph nodes, IL‐4 and IFN‐γ can perfuse broadly [125], while in tumours, IFN‐γ has been shown to activate bystander cells up to 800 μm from the source [122, 126].

Cytokine gradients structure the TME by regulating immune responses through diverse mechanisms. Produced by malignant, stromal and immune cells, cytokines such as interleukins, interferons, tumour necrosis factor (TNF) and transforming growth factors (TGF‐α and TGF‐β) coordinate inflammation, proliferation and cell death via receptor‐mediated signalling [120, 121]. Their diffusion can enhance antitumour immunity through bystander activation, where tumour‐reactive T cells release pro‐inflammatory cytokines that stimulate neighbouring noncancer‐antigen‐specific T cells [122, 127, 128]. The action of cytokines in the tumour context is complex and multifaceted. While cytokines such as IFN‐γ, IL‐2 and IL‐12 enhance antitumour immunity, and others such as TGF‐β typically promote immunosuppression, many, including IFN‐γ, exert dual roles depending on the context [120]. IFN‐γ can induce programmed death‐ligand 1 expression to promote immune evasion, but also upregulate MHC molecules, stimulate chemokine secretion (e.g., CXCL9 and CXCL10) and induce tumour‐cell death [129]. These effects are dose‐ and time‐dependent [130] and often modulated by the combinatorial cytokine milieu within the TME [80, 120, 122]. For instance, exposure to cytokines, such as IFN‐γ, IL‐12, TGF‐β, IL‐6, IL‐4 and IL‐2 results in mixed or intermediate CD4+ T‐cell phenotypes, rather than discrete polarised subsets, with TGF‐β exerting the strongest effect, followed by IL‐6 and IL‐4 in a hierarchical regulatory network [131].

### Molecular tumour patterning

Together, the molecular components of a tumour arrange in an intricate biochemical network that, through differential interactions with cancer cells and microenvironmental populations, patterns the developing tumour. A three‐dimensional analysis of colorectal cancers (CRCs) by Lin *et al*. revealed that in seemingly ‘chaotic’ tumours, the biochemical landscape regulates highly organised spatial architecture [18]. The authors identified opposing gradients of histone modifications, such as H3K27ac and H3K27me3, across tumour regions, defining zones of gene activation and silencing. Tumour morphology, including invasive fronts characterised by EMT, correlated not with single markers but with hyperdimensional features involving combinations of epigenetic regulators, oncogenes, cytokines and nutrients. A complementary study found distinct transcriptional programmes in tumour centres versus tumour–stroma interfaces across various cancer types [132]. Centre‐enriched genes supported protein synthesis and growth, while the periphery showed genes linked to invasion and immune evasion, including enolase 1 (*ENO1*), a glycolysis‐associated oncoprotein promoting invasion and immune suppression [133], as well as interferon‐stimulated gene 15 (*ISG15*), which supports immunosuppressive behaviour of macrophages [134].

Collectively, these findings demonstrate that cancer progression is not a random process but rather follows coordinated transitions. Lin *et al*. compared their observations to the morphogenetic fields structuring embryonic development, where cells differentiate into specialised types based on their position within a molecular gradient of extracellular cues [18, 81]. Both the biochemical landscape and physical forces influence tumour initiation and progression by shaping cellular behaviour, offering not just a scaffold but an active regulatory layer in tumour evolution.

## Cellular complexity in context: From single cells to tissue niches

### Tumour cellular heterogeneity and configuration

The composition and spatial arrangement of cells within tumours profoundly influence cancer progression, immune evasion and cancer therapy [3, 135, 136, 137]. Physical constraints and biochemical gradients converge at the cellular level, modulating cell‐fate decisions, driving phenotypic plasticity and orchestrating the behaviour of both malignant and nonmalignant cells [138]. Through these mechanisms, spatial organisation becomes not merely a feature of tumour structure, but a fundamental determinant of its function and evolutionary trajectory.

At the cellular level, environmental stimuli are integrated through gene regulatory mechanisms, resulting in variability in protein expression among individual cells within a population. These differences give rise to spatially patterned gene expression, which correlates with functional and morphological heterogeneity, establishing a complex dynamic interplay with other elements of tissue architecture [18, 69, 70, 71, 72, 73, 76, 77, 78, 79, 132, 139, 140].

The phenotypic plasticity inherent in cells, that is their ability to adapt behaviour and identity in response to environmental cues [141, 142, 143], constitutes a central pillar of tumour patterning and compartmentalisation, enabling the emergence of spatially distinct subregions and niches which foster tumour evolution and diversification [141, 142, 143]. A striking example is the existence of transient hybrid states during EMT. In colorectal cancer, gradients of hybrid phenotypes localise to invasive fibrillar protrusions at the tumour edge, linking phenotypic plasticity directly to tissue architecture [18]. A complementary study demonstrated tumour compartmentalisation into functionally distinct zones enriched for protein synthesis and proliferation, as well as invasion and immune evasion [132]. These findings highlight how spatially regulated phenotypic plasticity drives functional heterogeneity, enabling distinct behavioural programmes to emerge across tumour regions.

**Fig. 3:**
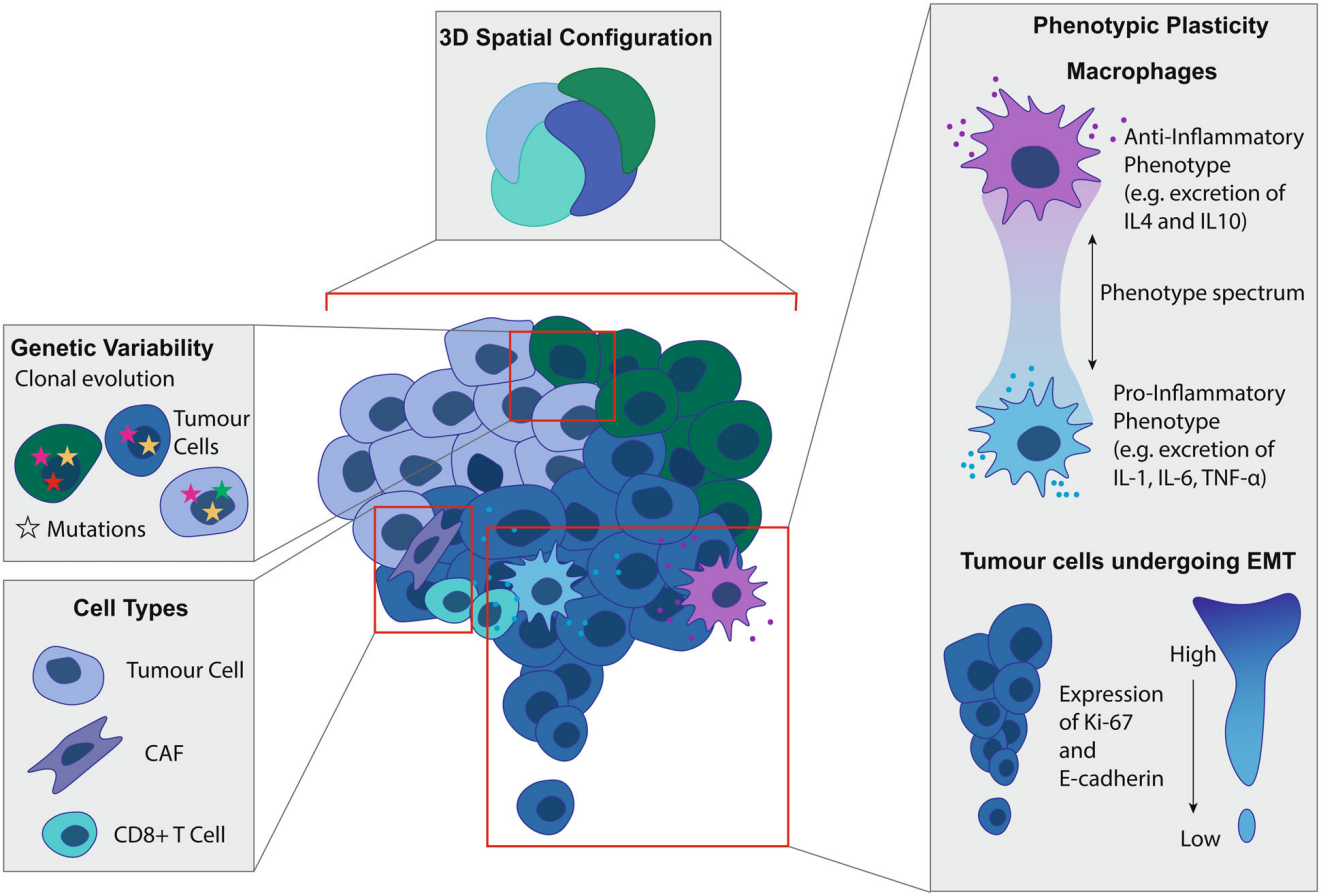
Cellular Heterogeneity and Configuration in Solid Tumours. Illustrative overview of the different aspects of cellular heterogeneity in solid tumours, depending on tissue architecture and encompassing genetic variability resulting from the accumulation of mutations and clonal evolution, diverse cell types within the tumour microenvironment and phenotypic plasticity driven by gene expression changes and 3D cellular configuration. CAF, cancer‐associated fibroblast; E‐cadherin, Epithelial cadherin; EMT, epithelial‐mesenchymal transition; IL4, interleukin 4; TGF‐ß, Transforming growth factor‐β; TNF‐α, Tumour necrosis factor alpha.

Importantly, phenotypic plasticity not only drives tumour evolution by diversifying cancer cell states but also extends to the surrounding microenvironment. A salient example is macrophage polarisation, wherein macrophages under the influence of cytokine gradients, mechanical forces and local metabolic cues adopt a range of immunomodulatory phenotypes. This process is orchestrated by signalling pathways, transcription factors and epigenetic mechanisms [72, 73, 144, 145]. Consequently, rather than existing in discrete M1 or M2 states, tumour‐associated macrophages occupy a continuum of activation profiles, contributing to immune suppression, tissue remodelling or inflammatory activation depending on their spatial and biochemical context (Fig. 3).

### The transforming context: How the cell of origin shapes tumour initiation

While multiple cells within a tissue can give rise to cancer, the specific cell of origin, defined by its unique chromatin and transcriptional states, influences the likelihood of tumour initiation and the phenotypic trajectory of the resulting malignancy [146, 147, 148, 149, 150, 151, 152]. For example, in breast cancer, single‐cell technologies have reinforced the link between subtype and cellular origin [149], supporting the widely accepted view that luminal A and B tumours likely arise from mature oestrogen receptor‐positive (ER+) luminal cells, while basal‐like breast cancers originate from luminal progenitor cells [153, 154]. These distinctions are underpinned by sustained transcriptional programmes and chromatin accessibility patterns. Basal‐like breast cancers exhibit increased chromatin accessibility for transcription factors, such as SRY‐box transcription factor 4 and 9 (SOX4 and SOX9), E2 promoter‐binding factor (E2F) family members, grainyhead‐like transcription factor 1 and 2 (GRHL1/2) and transcription factor CP2 (TFCP2), while luminal A and B tumours show increased chromatin accessibility for GATA binding protein 3 (GATA3), forkhead box protein P1 (FOXP1) and hepatocyte nuclear factor 1 alpha (HNF1A) [149].

**Fig. 4:**
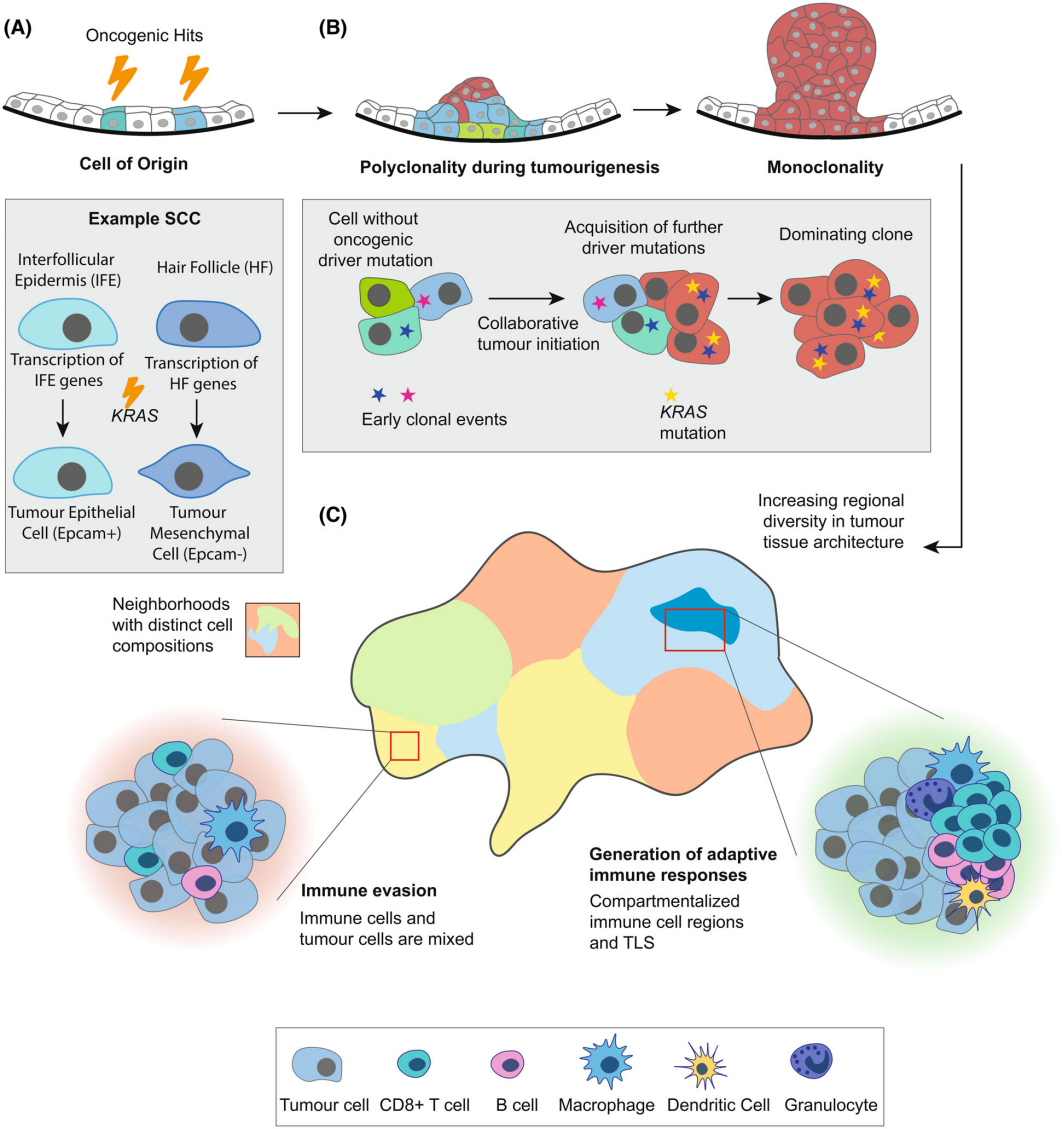
Cellular heterogeneity and 3D configuration during tumour initiation and progression. (A) The cell of origin's lineage‐specific gene expression influences tumour phenotypes derived from HF and IFE cells. HF‐derived tumour cells show a higher propensity for EMT, driven by the activation of EMT‐associated genes. IFE‐derived tumour cells remain differentiated, reflecting their distinct lineage‐specific transcriptional and epigenetic profiles. (B) Polyclonal tumour initiation arises from independent clones, including mutated and nonmutated cells, collaborating to overcome fitness barriers. Upon acquiring a strong driver mutation, such as KRAS, dominant clones outcompete others, resulting in monoclonality. (C) Tumour progression leads to increased heterogeneity in cell types, mutations and phenotypes, driven by environmental regional diversity. Cells organise into distinct 3D spatial arrangements and form cellular neighbourhoods, influencing patient prognosis and antitumour immunity. HF, hair follicle; IFE, interfollicular epidermis; KRAS, Kristen Rat Sarcoma Viral oncogene homologue; SCC, squamous cell carcinoma; TLS, tertiary lymphoid structure.

During tumour progression, the cell of origin retains specific chromatin states and transcriptional programmes, which are passed on and influence tumour evolution and subtype‐specific characteristics [146, 148, 149, 150, 152]. The cell of origin can impact tumour invasiveness by modulating the likelihood of undergoing EMT during progression [146, 148]. For instance, in SCCs, hair follicle‐derived tumour cells exhibit a higher propensity for EMT compared with interfollicular epidermis‐derived cells, which tend to remain well‐differentiated SCCs (Fig. 4A) [146]. This difference is due to the unique epigenetic and transcriptional landscapes of each lineage. hair follicle cells often possess EMT‐associated genes primed for activation, including transcription factors linked to stemness and differentiation, as well as signalling molecules that enhance TGF‐β signalling or inhibit bone morphogenetic protein (BMP) pathways. In contrast, interfollicular epidermis‐derived cells contain lineage‐specific factors, such as krüppel‐like factor 5 (KLF5) and tumour protein p63 (P63), which promote differentiated SCCs in response to oncogenic *Ras* expression and drive the expression of microRNAs like miR‐200, which target EMT transcription factors and downregulate EMT genes like Zinc Finger E‐Box Binding Homeobox 1 (*Zeb1*) [146].

### Tumour initiating propensity is shaped by Normal tissue architecture

The impact of tissue architecture on tumour‐cell behaviour precedes tumour initiation, with the establishment of epithelial regions differing in their tumorigenic potential. Stem cell niches, for example, organise epithelial compartments into progenitor cells and differentiated progeny. While differentiated cells have been shown to exhibit limited tumour initiating potential, adult tissue stem cells and progenitors, tasked with maintaining adult epithelia, are more vulnerable to transformation due to their intrinsic plasticity [155, 156]. Conversely, tumorigenesis from differentiated cells is often enabled through the hijacking of self‐renewal and differentiation pathways, with many oncogenic driver mutations targeting master developmental regulators or chromatin factors essential for cellular reprogramming [157, 158].

Although most tumours are understood to originate from a single progenitor cell (monoclonal origin), increasing evidence suggests that a significant proportion of tumours arise from multiple ancestral cells, referred to as polyclonal origin [159], as for example demonstrated in SCC [160] and CRC [161, 162, 163, 164, 165], where distinct cellular lineages expand concurrently within a single lesion. This alternative avenue relies on collaboration between multiple adjacent clones harbouring mutually beneficial genetic alterations to originate polyclonal tumours. In colorectal adenomas with adenomatous polyposis coli (*Apc*) mutations, clonal diversity circumvents fitness constraints through functional complementation among coexisting clones [164]. *Apc* loss of function in intestinal stem cells drives tumour initiation via aberrant WNT signalling, creating a permissive tumour environment [166, 167]. Polyclonal tumours often harbour independently arising *Apc* mutations and are sustained by spatially distinct stem cell populations corresponding to varying *Apc* mutational status, reflecting a dynamic and adaptable TME [164]. Polyclonal cooperation, together with the subclonal acquisition of additional mutations, such as in kirsten rat sarcoma viral oncogene homologue (KRAS) and MYC signalling cascades [168, 169], allows founder clones to compensate for pathway limitations, creating optimal conditions for tumour initiation and progression [164]. Importantly, polyclonal collaboration does not only involve oncogenic mutated cells but may also recruit epithelial cells lacking canonical driver mutations [160, 162]. Many neoplastic cells in premalignant colorectal glands lack canonical driver mutations, suggesting they may be influenced by neighbouring clones harbouring driver mutations [162]. Similarly, harvey rat sarcoma viral oncogene homologue (Hras)‐mutant clones in SCC actively recruit neighbouring epithelial cells to support tumour growth [160].

### Tumour progression

As tumours progress, tumour‐specific tissue architecture becomes increasingly dynamic and spatially compartmentalised, characterised by heterogeneity in physical forces, biochemical gradients and 3D cellular configurations [18, 20, 23, 139]. In progressing tumours, cancer cells exhibit genetic heterogeneity, arising through clonal evolution, where cells accumulate mutations and are subject to selective pressures based on their relative fitness within the surrounding environment [170]. As the tumour grows and more mutations accumulate, certain clones with particularly advantageous mutations (such as *KRAS* in CRC) dominate, driving the tumour towards a more monoclonal state (Fig. 4B) [162, 164]. However, as a tumour expands, regional differences in tissue architecture increase, creating distinct microenvironments that favour clonal diversification, leading to functional heterogeneity, even in a largely monoclonal tumour [18, 21, 170].

Clonal diversification fuels tumour evolution through spatially encoded competition and selection of tumour subclones that have genetically diversified since tumour initiation. This ongoing diversification refines tumour behaviour, enabling adaptation to changing host conditions and contributing to therapeutic resistance. Multiclonal crosstalk occurs within structured tissue compartments and is further shaped by the local recruitment and modulation of tumour‐associated stromal and immune cell populations. These niche interactions reinforce the spatial organisation of the tumour and guide the evolutionary dynamics of malignant clones.

### Cellular neighbourhoods and niches

Recent studies have highlighted conserved patterns in tumours, indicating underlying principles that guide cellular composition and configuration. These niches are defined by their biochemical milieu and the cellular neighbourhoods (CNs) that constitute them, and evidence from CRC suggests that their emergence may be largely conserved among patients [17]. CNs comprise transformed cells but also stromal cells, immune cells and endothelial cells [17, 18, 132] defined by their quantity, localisation and functional states of constituent cell types (Fig. 4C). Individual cells are affected by their neighbours; for instance, in breast cancer, close proximity between cancer cells undergoing EMT and macrophages in different functional states has been shown to promote metastasis [171, 172, 173].

Configurations of tumour‐cell immune cell interactions within CNs correlate with patient outcomes [17]. The spatial enrichment, rather than the total number of PD‐1 + CD4+ T cells within a granulocyte‐enriched CN was associated with improved survival, highlighting that the spatial positioning of immune cells within the tumour is more critical than their overall abundance [17]. In HPV‐negative head and neck squamous cell carcinoma (HNSCC), tumours with greater mixing of immune and tumour cells are associated with poorer progression‐free survival, while more compartmentalised tumours, where immune and tumour cells are spatially distinct, tend to have a denser immune cell organisation that supports antigen presentation and reduces immune suppression, fostering a more immune‐supportive environment [174]. Similarly, CRC patients with a Crohn's‐like reaction, marked by more organised immune responses, often have longer survival than high‐risk patients with diffuse inflammatory infiltrates, whose fragmented immune neighbourhoods and closer tumour‐immune interactions are associated with worse outcomes [17, 175, 176]. Overall, increased intermingling of immune and tumour cells allows tumour cells to directly suppress immune activity [18, 170, 174], and, across various cancers, patients with primary tumours that exhibit greater compartmentalisation between tumour cells and immune cell populations often demonstrate longer overall and/or progression‐free survival (Fig. 4C) [17, 140, 174, 177].

Exploring therapeutic strategies that disrupt adverse neighbourhood adaptations while promoting beneficial configurations could influence tumour evolution towards more favourable outcomes. Tertiary lymphoid structures (TLS) are configurations that have received increasing interest in recent years [178, 179]. TLS vary in maturity, ranging from simple B‐ and T‐cell aggregates to highly structured ectopic lymphoid organs resembling secondary lymphoid tissues, such as lymph nodes and spleen [178]. Mature TLS exhibit distinct T‐ and B‐cell zones, including germinal centre‐like regions where B cells clonally diversify and mature [180, 181]. These structures also include CD4+ and CD8+ T cells, dendritic cells, fibroblastic reticular cells and high endothelial venules, supporting continuous immune surveillance and response [182, 183, 184, 185]. This well‐structured spatial organisation within TLS is critical for fostering T‐ and B‐cell activation, proliferation and maturation, which has been repeatedly associated with improved patient prognosis across a wide range of cancers [17, 18, 140, 174, 175, 176, 177, 178, 181, 186, 187, 188, 189]. However, this association is context‐dependent and influenced by TLS maturity and cellular composition; in certain tumour types, such as gliomas, TLS presence has not been linked to improved survival, and their prognostic and therapeutic significance remains under investigation [190].

## Patient‐specific and systemic influences on tumour architecture

Beyond organ‐specific differences, tissue architecture is shaped by patient‐intrinsic considerations, including age, gender, body mass index, lifestyle, microbiome composition and pre‐existing conditions. Pathological conditions that alter tissue architecture, such as chronic inflammation in inflammatory bowel disease, can heighten cancer susceptibility [191, 192]. The wounding and repair inherent to inflammatory bowel disease impose selective pressure that favours mutant cells with enhanced rapid mucosal repair and resistance to inflammatory challenges [193]. These conditions remodel both the tissue architecture and the biochemical landscape, enriching it with DNA‐damaging agents such as ROS, which foster carcinogenesis. Furthermore, inflammatory cytokines and growth factors, including IL‐1, IL‐6, TGF‐β, IL‐17A and IL‐22, support tumour progression by promoting cell survival, proliferation and differentiation, processes that can be co‐opted by mutated cells [85].

## Conclusion and future perspectives

Tumour tissue architecture, comprising physical forces, biochemical gradients and spatial cellular organisation, is not merely a structural backdrop but a dynamic, integrative regulator of tumour behaviour. At the centre of this system lies the ability of cells to interpret and respond to a multitude of cues: mechanical stresses, such as stiffness, compression and shear; biochemical signals derived from gradients of oxygen, metabolites and cytokines; and interactions with neighbouring cells. These signals converge at the cellular level to drive phenotypic plasticity, enable cooperation among cell types and cancer cell clones, and ultimately shape tumour morphology, progression and immune evasion. Tumour heterogeneity is not simply stochastic; it is spatially patterned, structured by mechanical and biochemical gradients and reinforced by cell‐of‐origin constraints and evolutionary pressures. Recognising this spatial logic challenges reductionist models of cancer and reframes tumours as complex, organised ecosystems.

Consequently, our understanding of tumour biology is increasingly driven by the integration of spatial, mechanical and molecular analyses. The combinatorial complexity of molecular interactions within defined spatial and mechanical contexts continues to limit our capacity to fully decode tumour ecosystems. Multi‐modal single‐cell assays, spatial transcriptomics and high‐resolution 3D imaging technologies are beginning to reveal the architectural rules that underpin tumour behaviour. *In vitro* models such as assembloids and organ‐on‐chip systems hold promise for mechanistically dissecting how physical and biochemical cues modulate cancer cell fate and behaviour [194, 195, 196, 197, 198]. However, those assays remain limited in their ability to capture the dynamic and spatial complexity of tumour–host interactions observed *in vivo* and necessitate integration with advanced *in vivo* models to bridge the gap between experimental systems and biological complexity.

Recognising tumours as structured self‐organising systems carries therapeutic implications: Features such as vascular density, network structure and IFP impact anticancer drug delivery [199]. Yet, interventions must navigate tumoural structural complexity without disrupting beneficial compartments, such as antitumour immunity or normal tissue architecture, or inadvertently promoting more invasive configurations. The tumour–host interface, where cancer, immune and stromal cells converge, is a critical site of reciprocal instruction. Dissecting how ECM composition, mechanical confinement and immune cell topography co‐evolve at this interface could reveal new therapeutic entry points. Tumour architectural features are particularly critical in immunotherapy, where resistance mechanisms and the immunosuppressive nature of tumour tissue often hinder treatment success [179]. While immunotherapeutic efficacy is increasingly recognised to depend on the spatial and cellular tumour organisation, physical parameters, such as tissue stiffness, IFP and solid stress, also modulate immune cell infiltration, activation and cytotoxicity. Targeting these physical attributes may enhance immune accessibility and synergise with existing immunotherapies.

More fundamentally, decoupling the tightly interwoven physical and biochemical cues that underlie tumour aggressiveness represents a promising therapeutic avenue [138, 200]. Normalising tumour stiffness, decompressing vessels and modulating ECM composition may not only ablate the mechanotransducive signals that tumours exploit but also reconfigure the cellular landscape and render tumours more susceptible to chemotherapy and immune attack [67, 201, 202, 203].

## Author contributions

L.D., C.L. and H.A.M. were responsible for the writing of the original draft. S.P. reviewed the original manuscript. H.A.M. was responsible for the conceptualisation and supervision of the project.

## Conflict of interest

The authors declare no conflict of interest.
